# Peripheral venous congestion causes time‐ and dose‐dependent release of endothelin‐1 in humans

**DOI:** 10.14814/phy2.13118

**Published:** 2017-03-21

**Authors:** Jeffrey Lin, Neelesh Chudasama, Yacki Hayashi, Christopher Hawk, Sahadeo D. Ramnauth, Ka Yuk Wong, Ante Harxhi, Duygu Onat, Michiyori Wakabayashi, Nir Uriel, Ulrich P. Jorde, Thierry H. LeJemtel, Hani N. Sabbah, Ryan T. Demmer, Paolo C. Colombo

**Affiliations:** ^1^ Columbia University Medical CenterNew YorkNew York; ^2^ Tulane University School of MedicineNew OrleansLouisiana; ^3^ MedicineHenry Ford HospitalDetroitMichigan

**Keywords:** Congestive heart failure, endothelin, inflammation

## Abstract

Endothelin‐1 (ET‐1) is a pivotal mediator of vasoconstriction and inflammation in congestive states such as heart failure (HF) and chronic kidney disease (CKD). Whether peripheral venous congestion (VC) increases plasma ET‐1 at pressures commonly seen in HF and CKD patients is unknown. We seek to characterize whether peripheral VC promotes time‐ and dose‐dependent increases in plasma ET‐1 and whether these changes are sustained after decongestion. We used a randomized, cross‐over design in 20 healthy subjects (age 30 ± 7 years). To experimentally model VC, venous pressure was increased to either 15 or 30 mmHg (randomized at first visit) above baseline by inflating a cuff around the subject's dominant arm; the nondominant arm served as a noncongested control. We measured plasma ET‐1 at baseline, after 20, 60 and 120 min of VC, and finally at 180 min (60 min after cuff release and decongestion). Plasma ET‐1 progressively and significantly increased over 120 min in the congested arm relative to the control arm and to baseline values. This effect was dose‐dependent: ET‐1 increased by 45% and 100% at VC doses of 15 and 30 mmHg, respectively (*P *<* *0.05), and declined after 60 min of decongestion though remaining significantly elevated compared to baseline. In summary, peripheral VC causes time‐ and dose‐dependent increases in plasma ET‐1. Of note, the lower dose of 15 mmHg (more clinically relevant to HF and CKD patients) was sufficient to raise ET‐1. These findings support the potentially contributory, not merely consequential, role of VC in the pathophysiology of HF and CKD.

## Introduction

Endothelin‐1 (ET‐1) is an important vasoactive peptide primarily produced in vascular endothelial cells (ECs). In humans, its vasoconstrictor actions predominate with additional pro‐inflammatory, pro‐oxidant, thrombotic, mitogenic, and proliferative properties. As such, it has been implicated in the pathogenesis of congestive disease states such as heart failure (HF) and chronic kidney disease (CKD) (Komuro et al. 1988; Cowburn et al. 1999; Teerlink 2005).

Venous congestion and elevated plasma ET‐1 levels are frequently present in advanced and acute HF and kidney disease with a decline in levels with clinical improvement (Rodeheffer et al. 1992; Kohan 1997; White et al. 2006). While prognostic and pathophysiologic relevance of this increase in ET‐1 is widely accepted, causal mechanisms remain the subject of intense investigation.

We recently showed that plasma ET‐1 concentration and ET‐1 expression in venous ECs acutely increase in response to experimental severe venous congestion (VC) (Colombo et al. 2014). However, in this model, the increase in peripheral venous pressure (PVP) (which typically exceeds central venous pressure by 2–3 mmHg)(Munis et al. 2001; Hadimioglu et al. 2006) was extreme (30 mmHg above baseline levels) and short in duration (75 min), thus not realistically mimicking the hemodynamic conditions of peripheral congestion in HF and CKD patients. It is, therefore, critical to understand if lower levels of VC also lead to increased ET‐1 levels and if so how quickly these changes occur, and whether or not they are sustained in the setting of decongestion.

To address these scientific questions, we designed this study to test the hypothesis that acute experimental VC at levels similar to those clinically observed in congestive states is sufficient to cause a time‐ and dose‐dependent increase in local concentration of plasma ET‐1.

## Materials and Methods

We enrolled normotensive, nonsmoking subjects with no history of chronic illness or chronic medication use. Subjects had to abstain from alcohol consumption or exercise for at least 24 h prior to each test. The protocol was approved by the Institutional Review Board of Columbia University. All subjects provided informed written consent.

We used a randomized, cross‐over design to study two doses of experimental VC (15 or 30 mmHg, randomly assigned at the first of two encounters). Encounters were separated by at least 6 days.

After an overnight fast, subjects were placed in a quiet room, in a supine position for 30 min prior to initiation of the test. An 18‐gauge angiocatheter was placed in the antecubital or basilic vein of each arm. Local VC was then applied at either 15 or 30 mmHg above baseline level by inflating a cuff around the upper portion of the dominant arm (test arm). During the test, a pressure transducer (Agilent, Santa Clara, CA) was connected to the angiocatheter of the test arm to monitor PVP. Blood was sampled from the antecubital or basilic vein of the control arm at baseline (time 0) and from the control and test arms after 20, 60, and 120 min of VC and again at 180 min (i.e., 60 min after cuff release and decongestion). Vital signs and peripheral arterial oximetry were measured at baseline and after 120 min of VC.

Plasma ET‐1 was measured using a quantitative immunoassay kit (R&D Systems, Minneapolis, MN). Venous lactate was determined using an enzymatic colorimetric method on the Cobas Integra 400 Plus Analyzer (Roche Diagnostics, Indianapolis, IN). Results are expressed as the mean of duplicate determinations and values.

We used paired t‐tests to examine whether ET‐1 levels differed by time and/or level of VC.

## Results

Twenty subjects were studied. Nineteen completed the two‐encounter protocol. One subject was lost to follow up after the first encounter. Thus, the 15 mmHg data set includes 20 subjects, whereas the 30 mmHg data set includes 19 subjects. No adverse events were observed. Overall, study subjects were young and had a normal body mass index, lipid profile, serum glucose, and renal function (see Table** 1).

**Table 1:** Subject characteristics (*N* = 20)

Variable	Mean ± SD
Age (years)	30 ± 7
Gender (no. male/female)	19/1
Body mass index (kg/m^2^)	22.2 ± 2.9
Total cholesterol (mg/dL)	148 ± 26
Low‐density cholesterol (mg/dL)	79 ± 23
High‐density cholesterol (mg/dL)	55 ± 18
Triglycerides (mg/dL)	72 ± 41
Blood glucose (mg/dL)	94 ± 8
Serum creatinine (mg/dL)	0.86 ± 0.13

PVP rose from 4 ± 2 mmHg and 3 ± 2 mmHg at baseline to 19 ± 2 mmHg (*P* < 0.0001) and 33 ± 2 mmHg (*P* < 0.0001) at a VC dose of 15 and 30 mmHg, respectively.

Systolic blood pressure did not change in response to VC when compared with baseline (113 ± 9 vs. 113 ± 8 mmHg and 114 ± 6 vs. 117 ± 9 mmHg at a VC dose of 15 and 30 mmHg, respectively, all *P *= NS). Diastolic BP also remained similar (72 ± 6 vs. 73 ± 8 mmHg and 71 ± 6 vs. 76 ± 8 mmHg at a VC dose of 15 and 30 mmHg, respectively, all *P *= NS). Heart rate was lower after 120 min of VC when compared to baseline at a VC dose of 15 mmHg (65 ± 8 vs. 59 ± 7 bpm, *P *<* *0.01), but not at a VC dose of 30 mmHg (64 ± 9 vs. 60 ± 6 bpm, *P *= NS). Peripheral arterial oxygen saturation was 98 ± 1% at baseline and remained unchanged in response to both doses of VC.

In the congested arm, venous lactate was higher at baseline than after 120 min of VC though this difference was not significant (0.97 ± 0.34 vs. 0.84 ± 0.13 mmol/L and 0.86 ± 0.19 vs. 0.84 ± 0.14 mmol/L, at a VC dose of 15 and 30 mmHg, respectively, all *P *= NS). However, levels were higher in the congested arm when compared to control arm at both VC doses after 120 min (0.84 ± 0.13 vs. 0.74 ± 0.13 mmol/L and 0.84 ± 0.14 vs. 0.75 ± 0.19 mmol/L, at 15 and 30 mmHg, respectively, all *P *<* *0.05). Of note, all venous lactate results (range 0.50–1.21 mmol/L) were within normal limits (normal range: 0.56–2.22 mmol/L), and the lactate difference between congested and control arm at 120 min was similar with both VC doses (0.09 ± 0.09 and 0.09 ± 0.82 mmol/L, at a VC dose of 15 and 30 mmHg, respectively, *P *= NS).

**Figure 1:**
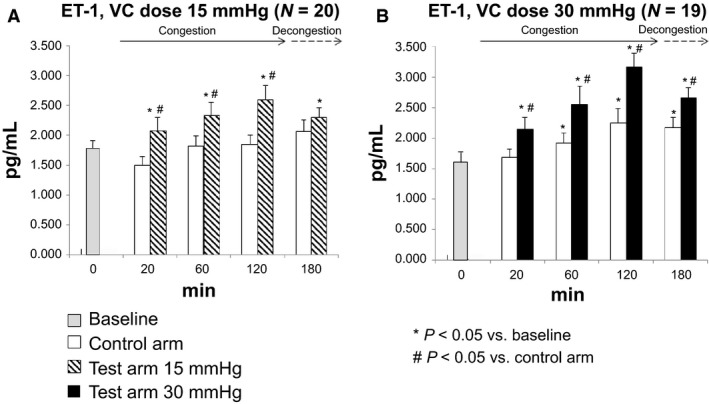
Plasma endothelin‐1 time response to venous congestion (VC) at a dose of 15 mmHg (A) and 30 mmHg (B); 30 mmHg data at 180 min in the test arm are based on a sample of 18 subjects; 15 mmHg data at 60 min in the control arm are based on a sample of 19 subjects. * *P* < 0.05 versus baseline; # *P* < 0.05 versus control arm.

Figure 1 shows plasma ET‐1 levels at a VC dose of 15 mmHg (*1A*) and 30 mmHg (*1B*) as a function of time. In the congested arm, both doses of VC were associated with a time‐dependent increase in plasma ET‐1, starting at 20 min, compared to baseline and control arm values. In the control arm, plasma ET‐1 increased significantly compared to baseline, only at a dose of 30 mmHg and after 60 min of VC. At both doses of VC, plasma ET‐1 decreased following decongestion, but remained elevated after 60 min compared to baseline.

**Figure 2:**
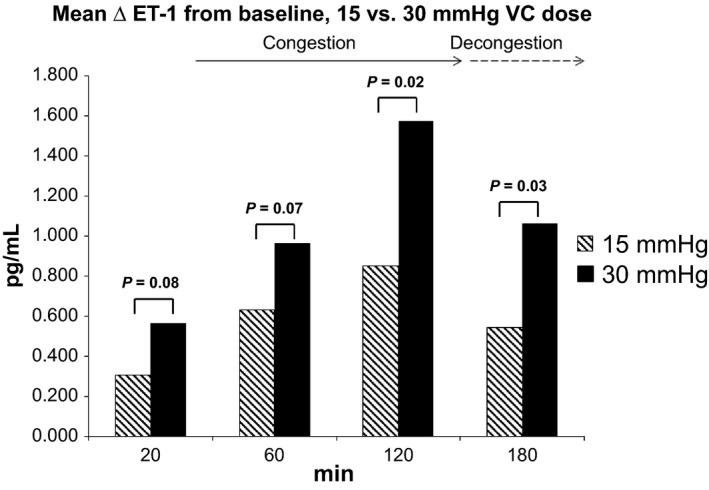
Plasma endothelin‐1 changes from baseline according to time and venous congestion (VC) dose. Change is computed as endothelin‐1 differences between each time point and baseline levels in the test arm; 30 mmHg data at 180 min in the test arm are based on a sample of 18 subjects; 15 mmHg data at 60 min in the control arm are based on a sample of 19 subjects.

Figure 2 compares the mean difference in plasma ET‐1 between each time point and baseline values in the congested arm for the two doses of VC. The increase in ET‐1 was consistently higher at a VC dose of 30 mmHg than 15 mmHg, though this was only statistically significant after 120 min of VC. The difference in plasma ET‐1 concentration between the two dosages remained significant after 60 min of decongestion.

## Discussion

Experimental VC causes a time‐ and dose‐dependent increase of ET‐1 with levels that decline, but remain elevated after 1 hour of decongestion. We previously showed that plasma ET‐1 levels increase after 75 min of extreme VC, 30 mmHg above baseline levels (Colombo et al. 2014). We now demonstrate for the first time that (1) lower, more clinically relevant levels of peripheral VC are sufficient to promote an acute increase in plasma ET‐1, (2) this increase is time dependent, and (3) plasma levels decline but remain elevated after 1 hour of decongestion.

Venous congestion is typically considered a consequence of HF and CKD. However, there is growing evidence that congestion itself may drive the pathogenesis of these chronic diseases as well. Recent work demonstrated that peripheral venous distension causes a sympathoexcitatory reflex leading to vasoconstriction and increases in systemic blood pressure (Cui et al. 2012). In our experimental model, clinically relevant levels of peripheral VC are associated with a local increase in plasma ET‐1. Plasma ET‐1 increases in patients with symptomatic HF and CKD. In HF patients, plasma levels rise to 2.1 ± 1.0, 2.6 ± 0.9, and 3.4 ± 0.8 pg/mL with increasing NYHA symptom class (I–III, respectively) (Kinugawa et al. 2003), while in patients with proteinuric CKD, the plasma levels of ET‐1 increase to reported levels of 3.6 ± 0.5 pg/mL (Dhaun et al. 2013). Importantly, these ET‐1 levels are similar to the those observed in our experimental model.

Interestingly, plasma ET‐1 also increased significantly in the control arm of the 30 mmHg group after 60 min of VC. The reason for this increase is not clear. Spillover from the test arm and systemic sympathoexcitatory reflex (Cui et al. 2012) are potential contributors to this finding.

Our current data do not fully elucidate the mechanisms for the increase in plasma ET‐1 in response to differing doses of peripheral VC. This increase could be the result of augmented ET‐1 production or be due to diminished ET‐1 clearance. Prior work investigating elevated ET‐1 levels in patients with congestive HF suggests that increases in ET‐1 are largely attributable to increased peptide production. In fact, the clearance rate of ET‐1 in these patients was noted to be increased (Parker and Thiessen 2004). Mechanistically, augmented production of ET‐1 secondary to venous distension is a plausible contributor. A prior study suggests that biomechanical stress in cultured endothelial cells upregulates the production of ET‐1 (Hasdai et al. 1997). Additional in vivo work in humans demonstrated that local venous congestion leads to differential expression of ET‐1 in venous endothelial cells (Colombo et al. 2014). Based on these prior results, it is reasonable to hypothesize that a local increase in endothelial ET‐1 expression and production may, at least in part, contribute to the observed increase in plasma levels of ET‐1 seen in this study.

It is important to note that tissue hypoxia may also promote endothelial ET‐1 production (Kourembanas et al. 1991). This is an important consideration in the current experimental model given the application of a pressure cuff in the test arm that may partially decrease arterial inflow in addition to venous outflow. Experimental VC did induce a marginal but significant dose‐independent increase in venous lactate in the congested arm when compared with the control arm, while postcongestion levels did not differ from baseline values, as previously reported (Colombo et al. 2014). However, (1) arterial oxygen saturation remained unchanged in response to both doses of VC; (2) all lactate levels were within normal limits; and, most importantly, (3) plasma ET‐1 increase was dependent on the VC dose while the difference in venous lactate between test and control arm at 120 min was the same with both doses of VC (i.e., dose‐independent). Taken together these results suggest that vascular stretch from VC rather than a marginal shift to anaerobic metabolism was primarily responsible for the observed increase in plasma ET‐1.

Finally, plasma levels of ET‐1 rapidly started to decrease during decongestion. To date, there is no clinical evidence that endothelin receptor antagonism leads to improved clinical outcomes in patients with decompensated HF undergoing concurrent decongestion with intravenous diuretics (McMurray et al. 2007). Results from this study suggest the provocative concept that decongestion may be sufficient in reducing the pathophysiologic effects of ET‐1 in severe congestive states, by decreasing plasma levels of ET‐1. Thus, endothelin receptor antagonism may be somehow redundant when coupled with adequate venous decongestion in this patient population.

Several limitations of this study set the stage for future work. First, women were not represented in our cohort of subjects. This is an important consideration given potential gender differences in endothelin biology (Ergul et al. 1998). Second, in contrast to our previous model which coupled blood and endothelial samplings, we did not pursue endothelial collections in this study, as we considered this approach (a total of five endothelial samplings per encounter) too high risk for the development of thrombophlebitis. Third, as mentioned previously, cuff inflation not only promoted VC but may have necessarily caused (1) a reduction in arterial perfusion pressure by impinging on the brachial artery and (2) hydrostatic pooling by reducing venous flow. Interestingly, these features are typical of advanced HF where arterial blood pressure progressively declines and chronic venous insufficiency is frequent (White and Ryjewski 2005), thus making our model even more relevant from a pathophysiological standpoint. Finally, we studied healthy subjects rather than patients with HF and/or CKD. However, this provides a strong proof‐of‐principle for the concept of hypervolemia as a fundamental stimulus for the pathophysiology of HF and CKD without confounding bias from comorbidities and/or medical therapies.

In conclusion, our present findings expand the concept that VC may be a contributor to as well as a consequence of the pathophysiology of congestive states such as HF and CKD through a release of ET‐1 that is time and dose dependent in nature. Our results also suggest that decongestion therapy and volume optimization may not only relieve symptoms, but also contribute to reduce ET‐1 mediated pathophysiological processes such as inflammation and vasoconstriction.

## Conflict of Interest

None.

## References

[phy213118-bib-0001] Colombo, P. C. , D. Onat , A. Harxhi , R. T. Demmer , Y. Hayashi , S. Jelic , et al. 2014 Peripheral venous congestion causes inflammation, neurohormonal, and endothelial cell activation. Eur. Heart J. 35:448–454. 24265434 10.1093/eurheartj/eht456 PMC3924182

[phy213118-bib-0002] Cowburn, P. J. , J. G. Cleland , J. D. McArthur , M. R. MacLean , J. J. McMurray , H. J. Dargie , et al. 1999 Endothelin B receptors are functionally important in mediating vasoconstriction in the systemic circulation in patients with left ventricular systolic dysfunction. J. Am. Coll. Cardiol. 33:932–938. 10091818 10.1016/s0735-1097(98)00663-9

[phy213118-bib-0003] Cui, J. , P. M. McQuillan , C. Blaha , A. R. Kunselman , and L. I. Sinoway . 2012 Limb venous distension evokes sympathetic activation via stimulation of the limb afferents in humans. Am. J. Physiol. Heart Circ. Physiol. 303:H457–H463. 22707559 10.1152/ajpheart.00236.2012 PMC3423143

[phy213118-bib-0004] Dhaun, N. , V. Melville , S. Blackwell , D. K. Talwar , N. R. Johnston , J. Goddard , et al. 2013 Endothelin‐A receptor antagonism modifies cardiovascular risk factors in CKD. J. Am. Soc. Nephrol. 24:31–36. 23243212 10.1681/ASN.2012040355 PMC3537212

[phy213118-bib-0005] Ergul, A. , K. Shoemaker , D. Puett , and R. L. Tackett . 1998 Gender differences in the expression of endothelin receptors in human saphenous veins in vitro. J. Pharmacol. Exp. Ther. 285:511–517. 9580591

[phy213118-bib-0006] Hadimioglu, N. , Z. Ertug , A. Yegin , S. Sanli , A. Gurkan , and A. Demirbas . 2006 Correlation of peripheral venous pressure and central venous pressure in kidney recipients. Transpl. Proc. 38:440–442. 10.1016/j.transproceed.2005.12.057 16549142

[phy213118-bib-0007] Hasdai, D. , D. J. Jr Holmes , K. N. Garratt , W. D. Edwards , and A. Lerman . 1997 Mechanical pressure and stretch release endothelin‐1 from human atherosclerotic coronary arteries in vivo. Circulation 95:357–362. 9008449 10.1161/01.cir.95.2.357

[phy213118-bib-0008] Kinugawa, T. , M. Kato , K. Ogino , S. Osaki , O. Igawa , I. Hisatome , et al. 2003 Plasma endothelin‐1 levels and clinical correlates in patients with chronic heart failure. J. Card. Fail. 9:318–324. 13680553 10.1054/jcaf.2003.39

[phy213118-bib-0009] Kohan, D. E. 1997 Endothelins in the normal and diseased kidney. Am. J. Kidney Dis. 29:2–26. 9002526 10.1016/s0272-6386(97)90004-4

[phy213118-bib-0010] Komuro, I. , H. Kurihara , T. Sugiyama , M. Yoshizumi , F. Takaku , and Y. Yazaki . 1988 Endothelin stimulates c‐fos and c‐myc expression and proliferation of vascular smooth muscle cells. FEBS Lett. 238:249–252. 3139457 10.1016/0014-5793(88)80489-7

[phy213118-bib-0011] Kourembanas, S. , P. A. Marsden , L. P. McQuillan , and D. V. Faller . 1991 Hypoxia induces endothelin gene expression and secretion in cultured human endothelium. J. Clin. Invest. 88:1054–1057. 1885767 10.1172/JCI115367 PMC295521

[phy213118-bib-0012] McMurray, J. J. , J. R. Teerlink , G. Cotter , R. C. Bourge , J. G. Cleland , G. Jondeau , et al. 2007 Effects of tezosentan on symptoms and clinical outcomes in patients with acute heart failure: the VERITAS randomized controlled trials. JAMA 298:2009–2019. 17986694 10.1001/jama.298.17.2009

[phy213118-bib-0013] Munis, J. R. , S. Bhatia , and L. J. Lozada . 2001 Peripheral venous pressure as a hemodynamic variable in neurosurgical patients. Anesth. Analg. 92:172–179. 11133622 10.1097/00000539-200101000-00033

[phy213118-bib-0014] Parker, J. D. , and J. J. Thiessen . 2004 Increased endothelin‐1 production in patients with chronic heart failure. Am. J. Physiol. Heart Circ. Physiol. 286:H1141. 14766679 10.1152/ajpheart.00239.2001

[phy213118-bib-0015] Rodeheffer, R. J. , A. Lerman , D. M. Heublein , and J. C. Jr Burnett . 1992 Increased plasma concentrations of endothelin in congestive heart failure in humans. Mayo Clin. Proc. 67:719–724. 1434909 10.1016/s0025-6196(12)60795-2

[phy213118-bib-0016] Teerlink, J. R. 2005 Endothelins: pathophysiology and treatment implications in chronic heart failure. Curr. Heart Fail. Rep. 2:191–197. 16332312 10.1007/BF02696649

[phy213118-bib-0017] White, J. V. , and C. Ryjewski . 2005 Chronic venous insufficiency. Perspect. Vasc. Surg. Endovasc. Ther. 17:319–327. 16389426 10.1177/153100350501700406

[phy213118-bib-0018] White, M. , A. Ducharme , R. Ibrahim , L. Whittom , J. Lavoie , and M. C. Guertin . 2006 Increased systemic inflammation and oxidative stress in patients with worsening congestive heart failure: improvement after short‐term inotropic support. Clin. Sci. (Lond.) 110:483–489. 16402915 10.1042/CS20050317

